# A conceptual framework on pre- and post-displacement stressors: the case of Syrian refugees

**DOI:** 10.3389/fpubh.2024.1372334

**Published:** 2024-04-26

**Authors:** Sara A. Assaf, Iman Nuwayhid, Rima R. Habib

**Affiliations:** ^1^Department of Epidemiology and Population Health, Faculty of Health Sciences, American University of Beirut, Beirut, Lebanon; ^2^Department of Environmental Health, Faculty of Health Sciences, American University of Beirut, Beirut, Lebanon

**Keywords:** Syria, refugees, pre-displacement, post-displacement, stressors, war, framework, mental health

## Abstract

Researchers have documented multiple stressors and mental health problems along the journey of refugees as they are displaced to seek refuge in nearby and remote host countries. This article examines published research on Syrian refugees to propose a framework to conceptualize Syrian refugees’ pre- and post-stressors and their collective impact on their mental health. The proposed framework provides a comprehensive understanding of the interconnected pathways between pre-displacement stressors, post-displacement stressors, and mental health outcomes for Syrian refugees. Pre-displacement stressors are best captured by the concept of trauma centrality and emotional suppression. Post-displacement stressors, categorized under financial, political, and social themes, have a direct impact on the mental health of the refugees, but could also play a partial mediating role on the impact of pre-displacement stressors on mental health. The framework suggests a direct pathway between the experience of war-related traumatic events and mental health and introduces the country of residence as a potential moderator of the severity of mental health. The latter is primarily influenced by local policies and the host communities’ acceptance of refugees. We believe that the proposed framework can guide the work of researchers, policymakers, and practitioners concerned with the mental health and well-being of Syrian refugees. Additionally, although based on the experience of Syrian refugees, it presents a holistic perspective that could be adapted in other refugee settings.

## 1 Introduction

The global refugee crisis has reached an alarming peak, with a twofold increase in the number of individuals forcibly displaced over the past decade (1). As per recent data from the United Nations High Commissioner for Refugees (UNHCR), until mid-2023, approximately 110 million individuals globally experienced forced displacement, driven by factors including persecution, conflict, violence, and human rights violations (2). Notably, over half of all refugees (52%) under UNHCR’s mandate originate from just three countries listed in descending order: the Syrian Arab Republic, Afghanistan, and Ukraine (2).

Refugees face an increased risk of mental health disorders surpassing the prevalence observed in non-war-affected populations (3). Earlier research on the mental health of refugees often assumed that symptoms were mostly linked to pre-displacement war-related experiences, giving rise to a war exposure model of refugee distress. While research adopting this model generally supported a ‘dose–response effect’, with higher war exposure levels predicting increased distress or diagnosis likelihood (4, 5), the strength of this effect varied, and the model often failed to account for significant unexplained variance in mental health symptom levels (6–8). This variability was attributed to the exclusion of post-displacement stressors faced in exile, reflecting an underestimation of the psychological impact of displacement on mental health (9, 10). These stressors include social isolation, poverty, discrimination, uncertainty about asylum status, unemployment, poor housing conditions, and others (11, 12).

The subsequent ecological model of refugee distress, which also incorporates post-displacement stressors, proved more predictive than the earlier war exposure-focused model (13–15). In recent research, post-displacement stressors consistently predicted mental health disorder levels (12, 16–18), with numerous studies revealing that these stressors accounted for greater variance in depression and anxiety levels than war-related experiences of trauma and loss (14, 15, 19). Post-displacement stressors were also positively associated with post-traumatic stress disorder (PTSD) (20–22), possibly due to their traumatic nature and the depletion of coping resources in exile, rendering individuals more vulnerable to the effects of prior war exposure (23, 24).

While recent literature has broadened its scope to encompass post-displacement stressors in addition to traumatic events experienced prior to migration, there are still gaps in fully understanding and evaluating the intersectionality of pre- and post-displacement stressors on refugees’ health. Furthermore, there is a tendency to generalize the same stressors to all refugees without accounting for the unique contextual factors experienced by different refugee groups in various host countries.

Syrian refugees have attracted considerable scholarly attention due to their major contribution to the worldwide refugee crisis and their widespread presence across numerous host countries. Drawing on literature published on Syrian refugees, this article seeks to offer a comprehensive exploration of the displacement stressors. This includes war-related stressors, with a particular emphasis on post-displacement stressors encountered in exile countries. Additionally, it aims to propose a framework to conceptualize Syrian refugees’ pre- and post-displacement stressors and their collective impact on health.

## 2 The case of Syrian refugees

Even after more than a decade of the Syrian conflict, the Syrian refugee crisis continues to be one of the largest displacement and humanitarian crises globally (25). Following the war in Syria, which began in 2011, over 350,000 Syrians have lost their lives and over 14 million people have been forced to flee their homes in search of safety (26). Of these, nearly 6.8 million individuals remain internally displaced, and the rest seek asylum in over 130 countries (25). Neighboring countries bear the heaviest burden, with Turkey hosting the largest number of Syrian refugees (over 3.5 million), followed by Lebanon (over 814,000) and Jordan (over 660,000) (27). Germany is the largest Syrian refugee-hosting country in Europe (around 522,000 individuals). Other hosting countries by descending order include Iraq, Egypt, Sweden, Sudan, Austria, Netherlands, Greece, France, Bulgaria, Switzerland, Denmark, and many others (27). These figures only account for registered refugees under UNHCR and do not include the substantial number of unregistered refugees living in these nations (25, 28). Studies on mental health in adult Syrian refugees collectively show an increased susceptibility to mental illnesses, with potentially over tenfold higher likelihood of developing post-traumatic stress and other disorders compared to the general population in the host country (29). This increased risk is attributed to the substantial number and intensity of war traumatic events encountered, along with post-displacement stressors (29).

### 2.1 Pre-displacement stressors

#### 2.1.1 The overall impact

A preponderant body of literature addressed the impact of war-related stressors on the mental health of Syrian refugees. A recent systematic review found that Syrian refugees have encountered a notably high number of traumatic events (29), with the prevalence rates for experiencing war-related events surpassing those observed in other groups of forced migrants (29, 30). The association between traumatic events and mental health disorders has been highlighted by several systematic reviews (29, 31, 32), with the severity of PTSD symptoms increasing with the number of traumatic events experienced (33–36). The most commonly experienced traumatic events in this population, as reported by a recent systematic review (32), were, in sequential order: ‘living in a war-affected area; the experience of the death of someone close; experiencing a life-threatening accident; experiencing a life-threatening accident of someone close; experiencing the torture of someone close; experiencing the abduction or being taken hostage of someone close; experiencing torture; and experiencing someone else’s torture, beating, or sexual abuse’. Other significant pre-displacement stressors include difficulty meeting basic needs (37, 38) and forced displacement (39).

#### 2.1.2 The role of trauma centrality and emotional suppression

The impact of war-traumatic experiences on refugees’ mental health is depicted by a complex framework that shows how traumatic events, trauma centrality, emotional suppression, PTSD, and psychiatric comorbidity are interconnected (40). War trauma centrality arises when adverse events experienced during wars can introduce a turning point in refugees’ life course and create a traumatized identity (41). Emotional suppression is an individual’s ability to consciously restrain the expression of unpleasant emotions such as anger and anxiety, thereby keeping these emotions unresolved (42). A Swedish study of 564 Syrian refugees demonstrated that emotional suppression acts as a mediator between trauma centrality and psychiatric disorders (40).

### 2.2 Post-displacement stressors

#### 2.2.1 The overall impact

Most research addressing the experiences of Syrian refugees after their displacement has primarily focused on specific post-displacement stressors, while minimal attention was given to understanding their collective negative influence on the refugees’ mental health, specifically PTSD, depression, and anxiety. To our knowledge, only one systematic review has holistically evaluated the overall post-displacement stressors as predictors of Syrian refugees’ mental health (31). This review identified specific factors, such as unmet social support needs, economic difficulties, and unemployment, that are associated with higher levels of anxiety, depression, and PTSD (31). Few other reviews have assessed specific stressors experienced during exile as potential indicators of their mental health outcomes, yielding inconsistent findings regarding the impact of economic challenges (32), legal status (32), and settlement types (29, 32). In contrast, within the systematic review, eight studies examining the resettlement period as a predictor of mental health among refugees revealed no association. The mean duration of resettlement across these studies ranged from 6.5 months to 3.4 years (32, 43).

#### 2.2.2 Themes of exile stressors

To gain a better understanding of the specific post-displacement stressors and their types, multiple research studies were reviewed. In a mixed methods study among Syrian refugees in Jordan, Alfadhli and Drury reported a typology of 33 secondary stressors organized into three main themes: financial, environmental, and social (44). Financial constraints included loss of income and property, high living costs in exile, poor housing conditions, and insufficient access to healthcare and educational services (44). Environmental stressors arise from laws restricting the integration of these refugees into host countries, such as prohibitions on employment and legal status (44). Social stressors include social relationships with the Jordanian host community (prejudice and exploitation), safety concerns, and government-enforced discrimination (44). Similar patterns of post-displacement stressors were reported in Lebanon, Turkey, and Jordan, where refugees experienced poor living conditions (45), economic constraints (45–47), discrimination (45, 47), exploitation from the host community (45), feeling unsafe and unprotected (46), fears of being forced to return to Syria (47), and concerns about getting treatment for health problems (47). In the latter Jordanian study, out of 14 post-displacement stressors that were evaluated, it was found that each additional stressor was significantly associated with increased odds of mental health conditions, including 32% increased odds of depression, 28% increased odds of anxiety, and 46% increased odds of PTSD (47).

#### 2.2.3 Key stressors

It is worth noting though that the majority of publications focused on one specific post-displacement stressor and the pathway by which this particular stressor affects the refugees’ mental health. One important financial stressor is lower income which was directly correlated with higher PTSD symptoms among Syrian refugees in Germany (48). A study on perceived needs among in south-central Turkey in 2013 found that nearly three-quarters of the surveyed refugees identified ‘income or livelihood’ as one of their top three priorities (49).

Ethnic discrimination, a major social stressor in the context of Syrian refugees, was found to be significantly associated with higher levels of depression and anxiety (45, 50). Disrupted social networks are another significant stressor confronting Syrian refugees in exile. This point was demonstrated by a multi-centered study, through which lower social support levels were significantly correlated with higher levels of anxiety and depression (51). Conversely, higher levels of social capital and social cohesion were linked to improved emotional well-being among Syrian refugees in Lebanon (52). Loss of culture and support in the post-displacement environment was the most powerful, and the only consistent, predictor of mental health status among Syrian refugees in Turkey, even when pre-displacement factors were taken into account (53).

Political stressors, such as challenges in residence and work permits, also play a key role in predicting the mental health of Syrian refugees. In Germany, more severe symptoms of PTSD were significantly associated with shorter validity of the refugees’ residence permission (54). In another study in Greece, the majority reported inadequate or nonexistent access to legal information and assistance regarding asylum procedures, and the heightened uncertainty surrounding their status intensified their anxiety levels (55).

#### 2.2.4 Comparative analysis

Few articles in the literature discussed the differences in the impact of pre- and post-displacement stressors on the mental health of Syrian refugees, either directly or indirectly. Findings from hierarchical regression analyses in Turkish camps demonstrated that post-displacement living difficulties had a more substantial impact on mental health outcomes compared to pre-displacement traumatic events (53). When comparing the mental health of Syrian refugees in Turkey with internally displaced individuals in Syria, a study found a higher prevalence of major depressive disorder among Turkish refugees (56). Notably, post-displacement factors were identified as stronger predictors of depression and PTSD than pre-displacement events (56).

#### 2.2.5 How post-displacement stressors differ in host countries?

It has been argued that the type and severity of post-displacement stressors can vary considerably among countries, partly influenced by local policies governing the refugees’ residence, movement, and employment, as well as the acceptance of host communities toward refugees (22). Studies comparing the prevalence of mental disorders in Syrian refugees across different countries reveal that those who resettled in high income countries, such as Sweden or Germany, experience lower levels of PTSD (33), anxiety symptoms, and depressive symptoms (57) compared to those living in Turkey. In addition, higher prevalence rates of panic disorder, PTSD, and generalized anxiety disorder were found among internally displaced refugees in Syria than those in Turkey (56). This difference may be linked to the availability of better living conditions in Turkey, including secure and stable housing, access to clean water and sanitation, and a safer environment, as evidenced by 74.4% expressing satisfaction with their living conditions in Turkey (58). A multi-country study aimed at identifying and comparing self-reported post-displacement stressors among Syrian refugees in different settings found that more than half of the participants reported challenges linked to camp-related living difficulties (Jordan), financial challenges (Turkey), employment (Jordan and Switzerland), and government regulations such as temporary residency (Switzerland) (59).

## 3 The proposed framework

**Figure 1:**
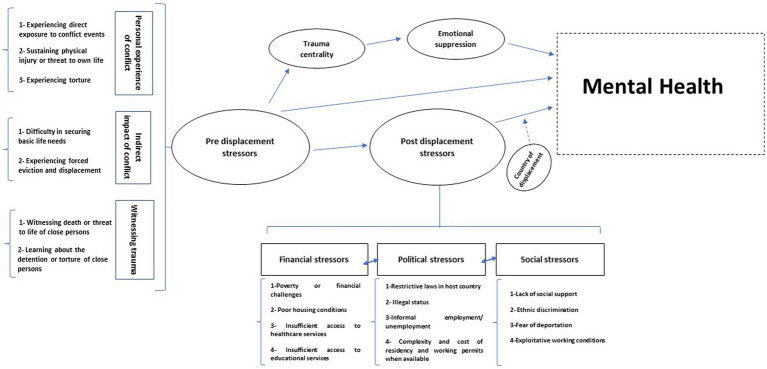
A theoretical framework describing the pre and post displacement stressors among Syrian refuges and their association with mental health. Dashed arrows represent a moderating effect.

Drawing from the existing literature on Syrian refugees, we propose a conceptual framework to depict the stressors encountered before and after displacement, along with their impact on their mental health (Figure 1). The preliminary Di-Acyclic Graphs (DAGs) corresponding to this figure are provided in Appendix A.

This framework includes the most frequently reported war trauma stressors as well as post-displacement stressors among Syrian refugees in various countries.

The framework presents a direct pathway between pre-displacement stressors and mental health. However, it integrates the trauma centrality concept to emphasize that war-related traumatic events may lead to a traumatized identity leading to mental health disorders, which are mediated by emotional suppression (33, 40–60). The importance of trauma centrality among refugees is especially apparent in war-related traumatic events (41). It is argued that for trauma to achieve centrality, it must be of high severity, serving as a pivotal turning and reference point in one’s life and becoming an integral part of their identity (41). For Syrian refugees, war trauma goes beyond being a mere memory; it integrates deeply into their sense of self, forming a stable traumatized self across various situations (60). These intense traumas disrupt personal reference points, influencing how individuals attribute meaning to their existing beliefs, feelings, experiences, and future expectations (60). The memories of war become a crucial turning point, reshaping their personal identity (60).

The framework also emphasizes that exposure to pre-displacement stressors exhibits an indirect effect on mental health, which is partially mediated by post-displacement stressors. This suggests that individuals who have fled conflict and face post-displacement life challenges are at an elevated risk of mental health disorders (61).

The framework also shows the direct and strong impact of post-displacement stressors on mental health (31–56). These stressors are categorized into financial, political, and social themes, which are suggested to be interlinked. Each theme can potentially predispose or exacerbate the others. For instance, the restrictive laws in host countries act as primary drivers compelling refugees to seek informal employment, often under exploitative working conditions be it disproportionately low wages, thus becoming a significant contributing factor to poverty (62, 63). Thus, political stressors can predispose both social and financial stressors.

Importantly, the framework identifies the country of residence as a factor that moderates the association between post-displacement stressors and mental health.

## 4 Discussion

Our proposed framework aligns with a few existing models that address the mental health of refugees, although they are not specifically tailored to Syrian refugees. In our framework, we emphasize the direct impact of pre-displacement stressors on refugees’ mental health, as well as an indirect impact partially mediated by post-displacement stressors. One of the existing frameworks presents an ecological model, which similarly indicates a direct impact of war exposure on mental health, along with an indirect impact partially mediated by post-displacement stressors (13). Another framework, centered on the Tamil refugees in Australia, suggests that war-related traumas directly affect Post-traumatic Symptoms (PTS) among refugees and also indirectly through specific post-displacement stressors (asylum difficulties, adaptation difficulties, and loss of culture and support) (61). However, the Tamil refugees model relied on data collected from 196 Tamil refugees living in Australia (61). Stressors excluded from the model, deemed non-impactful on PTS symptoms in this specific group, may still have a significant influence on PTS symptoms in diverse refugee populations and contexts.

In this framework, we posit that the impact of post-displacement stressors is major, sometimes exerting a more substantial influence on mental health compared to war-related stressors in the case of Syrian refugees. This implies that it is crucial to prioritize and thoroughly continuous stressors arising in exile when addressing and evaluating mental health concerns within this refugee population.

Furthermore, our framework highlighted the interconnectedness of post-displacement stressors. Thus, interventions targeting one specific stressor in exile can create a cascading effect, reducing the occurrence and intensity of other stressors and contributing to the mitigation of mental health issues in this refugee population. Additionally, it considers the country of residence as a crucial moderator influencing the severity of mental health among refugees. The moderation effect of the country of residence is primarily influenced by local policies and the host community’s acceptance of refugees which determine the types and severity of stressors in different host countries.

## 5 Implications and limitations

The conceptual framework proposed for understanding the mental health of Syrian refugees exhibits several notable strengths. First, it offers a comprehensive integration of the stressors experienced by refugees from war and throughout their settlement period. This inclusive approach enables a nuanced understanding of the complex interplay between various stressors and their cumulative impact on mental well-being. Second, the framework incorporates the concept of trauma centrality, shedding light on how war-related traumatic experiences can shape an individual’s self-perception and contribute to mental health disorders.

This framework combines theoretical foundations derived from an extensive literature review with the addition of practical statistical considerations. The pathways presented in the framework facilitate a clear visualization of the sequential relationships between stressors and mental health outcomes, enhancing its explanatory power and utility for researchers. It serves as a foundational resource for designing comprehensive data collection tools and guiding the data analysis process by identifying factors that should be considered as moderators. This is especially relevant in multicentered studies investigating the impact of specific post-displacement stressors on mental health in Syrian refugees, particularly in diverse settings such as high and low-income host countries.

In addition, the framework proposed partial mediating pathways through post-displacement stressors and trauma centrality. Accounting for mediators is essential in statistical analyses, as it helps disentangle and understand the underlying mechanisms that drive relationships between variables. Incorporating mediators facilitates identifying and controlling for intermediate factors, providing a more accurate depiction of the true associations within the data. This approach does not only enhance the robustness of statistical models but also contributes to the development of more informed and targeted interventions or policies based on a nuanced understanding of the studied phenomena.

To illustrate, this framework provides a solid foundation for developing effective and comprehensive humanitarian approaches addressing the mental health of refugees. The majority of mental health interventions among refugees rely on trauma-treatment protocols, cognitive-behavioral methods (64), and psychiatric medications (65) to treat PTSD and depression, which are believed to stem from war exposure. The variability in the effectiveness of such interventions is mainly linked to the failure to address exile-related stressors such as poverty, unemployment, social isolation, and precarious housing conditions (66, 67). Consequently, comprehensive approaches addressing the impact of prior war exposure and ongoing stressors, appear to hold considerable treatment potential for refugees who have access to clinic-based mental health services. Additionally, in contexts where psychotherapeutic interventions are scarce, humanitarian projects targeting livelihoods, policy changes to support refugee employment and resettlement, and the creation of supportive environments would enhance refugees’ mental health, even if not originally designed for this purpose. This has been demonstrated among displaced Rohingya adults, where access to employment opportunities and sufficient humanitarian aid have been identified as potential interventions to reduce the high prevalence of severe post-traumatic stress symptoms (PTSSs) (68).

Despite these strengths, the conceptual framework has a few limitations. Firstly, due to the emphasis on clarity and model simplicity, the framework did not incorporate all positive factors that could potentially act as buffers against displacement stressors on refugees’ mental health. This exclusion notably includes community-level interventions, such as aid organizations, which may play a role in mitigating ongoing stressors’ impact. Secondly, the framework did not include coping strategies and buffering factors like financial support, social networks, and cultural identity, which are known to influence resilience and mental well-being post-displacement. Lastly, while the framework suggests that ongoing post-displacement stressors may have a greater impact on mental health compared to war-related stressors, this assertion is based on limited empirical evidence, highlighting the need for further research to validate and expand upon these findings.

In conclusion, the proposed model introduces an innovative approach to conceptualizing the pre- and post-displacement stressors encountered by Syrian refugees and their impact on mental health. Although derived from literature focused on Syrian refugees, we posit that it can be adapted to other refugee populations. Considering the substantial size of the Syrian refugee population and their widespread distribution across various host countries, their diverse experiences in exile often mirror those of other refugees also displaced by war (2). Consequently, this framework could serve as a comprehensive foundation for researchers, policymakers, and practitioners interested in the health and well-being of refugees in general.

## Data availability statement

The original contributions presented in the study are included in the article/Supplementary material, further inquiries can be directed to the corresponding authors.

## Author contributions

SA: Conceptualization, Writing – original draft, Methodology, Visualization, Writing – review & editing. IN: Funding acquisition, Methodology, Supervision, Visualization, Writing – review & editing. RH: Funding acquisition, Methodology, Supervision, Visualization, Writing – review & editing.
