# Supplementary material for: A conceptual framework on pre- and post-displacement stressors: the case of Syrian refugees
Source: Front Public Health. 2024 Apr 26;12:1372334. doi: 10.3389/fpubh.2024.1372334 (PMC11082271; doi:10.3389/fpubh.2024.1372334)
Supplement: Supplementary file 1 [file Data_Sheet_1.docx]

# Appendix A:


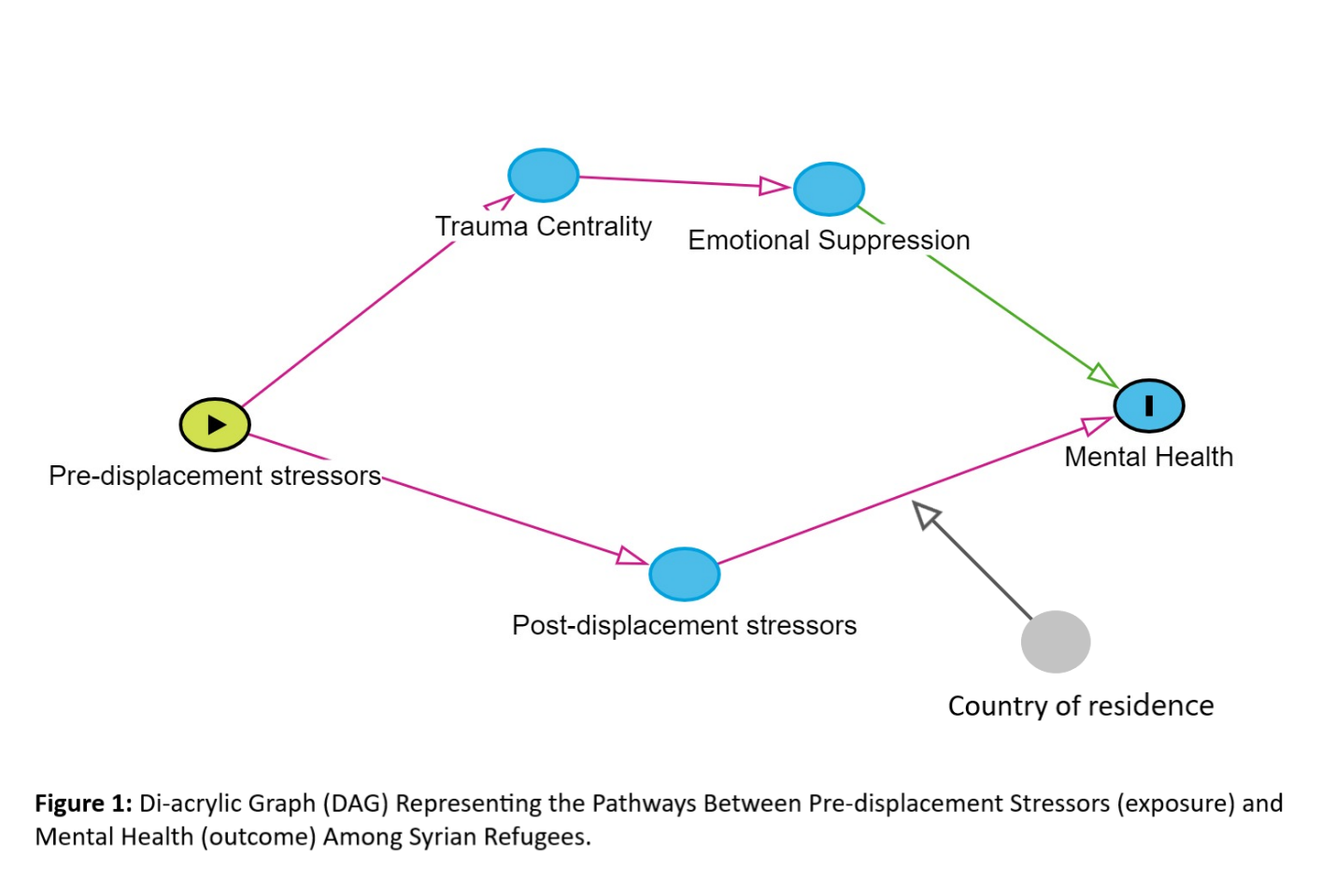


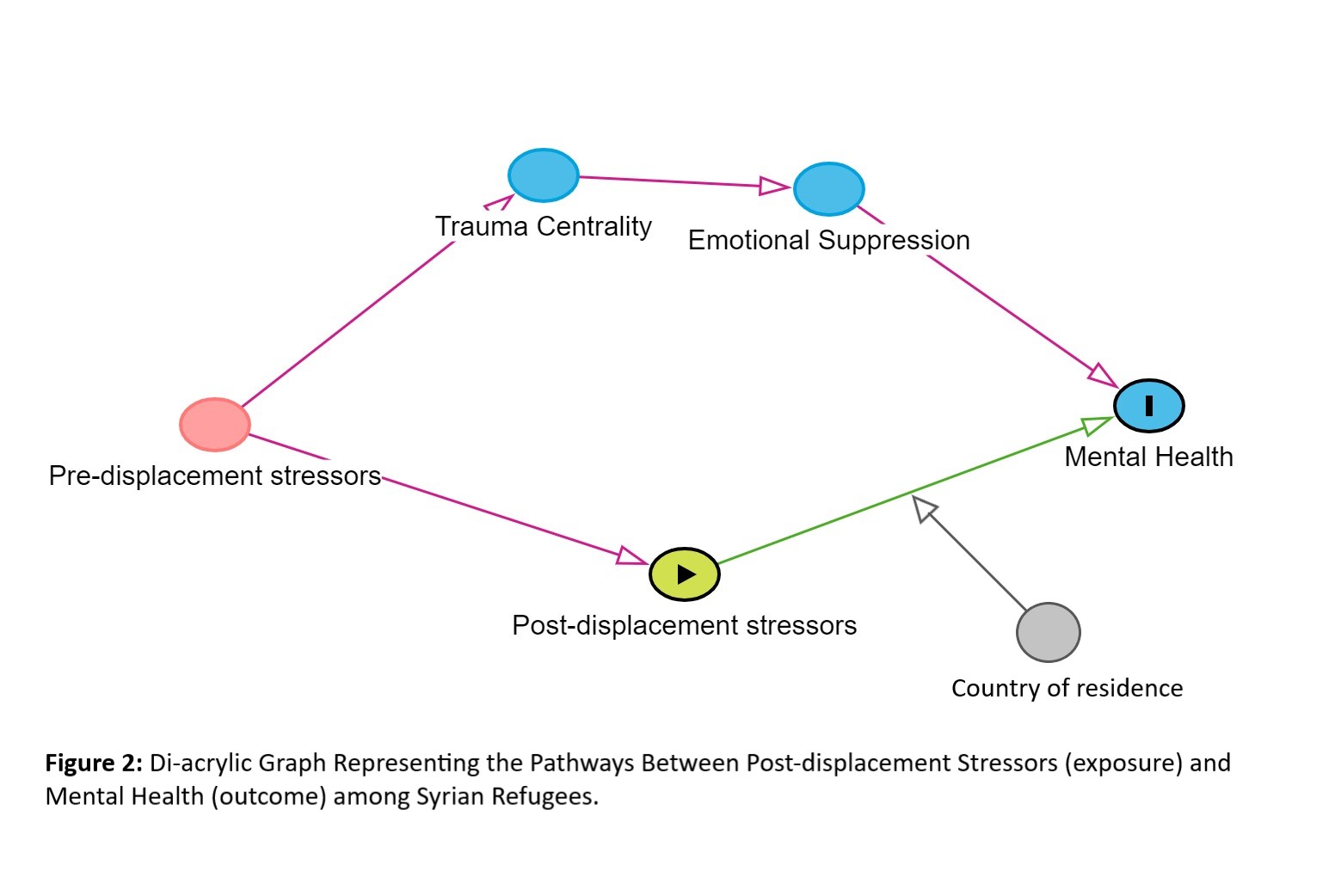


**Key:**


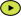
 exposure


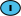
 outcome


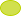
 ancestor of exposure


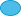
 ancestor of outcome

 ancestor of exposure *and* outcome


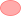


moderator


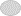


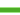
 causal path

- biasing path


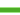
 moderating pathway*

* Country of displacement acts as a moderator on the association between post displacement stressors and refugees’ mental health:

**Detailed Explanation:**

**In Figure 1: Direct Effect Model**

**Exposure:** Pre-displacement stressors

**Outcome:** Mental Health

**Minimal sufficient adjustment sets for estimating the direct effect of Pre-displacement stressors on Mental Health:**

- Emotional Suppression, Post-displacement stressors
- Post-displacement stressors, Trauma Centrality

**In Figure 2: Direct Effect Model**

**Exposure:** Post-displacement stressors

**Outcome:** Mental Health

**Biasing paths are open.**

**Minimal sufficient adjustment sets for estimating the direct effect of post-displacement stressors on Mental Health:**

- Pre-displacement stressors
- Country or residence as a moderator

**Note**: These Di-Acyclic Graphs (DAGs) are based on available knowledge in the current literature. Further research is needed to incorporate specific confounders and stressors of interest.
